# Validation of the Italian version of the Questionnaire for Impulsive-Compulsive Disorders in Parkinson’s Disease-Rating Scale (QUIP-RS) in an Italian Parkinson’s disease cohort

**DOI:** 10.1007/s10072-024-07304-2

**Published:** 2024-01-17

**Authors:** Gianpaolo Maggi, Carmine Vitale, Chiara Giacobbe, Angelo Barone, Clara Mastromarino, Federica Iannotta, Marianna Amboni, Daniel Weintraub, Gabriella Santangelo

**Affiliations:** 1 Department of Psychology, University of Campania “Luigi Vanvitelli”, Caserta, Italy; 2 Department of Advanced Medical and Surgical Sciences, University of Campania “Luigi Vanvitelli”, Naples, Italy; 3 Department of Medical, Motor Sciences and Wellness, University “Parthenope”, Naples, Italy; 4 Institute of Diagnosis and Health, IDC-Hermitage Capodimonte, Naples, Italy; 5 Department of Neuroscience, Section of Psychiatry, School of Medicine, University Federico II, Naples, Italy; 6 Department of Medicine, Surgery and Dentistry, University of Salerno, Salerno, Italy; 7 Department of Psychiatry, Perelman School of Medicine at the University of Pennsylvania, Philadelphia, PA USA

**Keywords:** QUIP, Parkinson’s disease, Impulse control disorder, Impulsivity, Rating scale, Validation

## Abstract

**Introduction:**

Impulse control disorders (ICDs) frequently occur in Parkinson’s disease (PD), and an early identification is essential to prevent severe psychosocial consequences. The Questionnaire for Impulsive-Compulsive Disorders in Parkinson’s Disease–Rating Scale (QUIP-RS) has been developed to evaluate the severity of ICDs along with a range of impulsive-compulsive behaviors (ICBs) in PD; however, its Italian version has not yet been validated.

**Methods:**

One hundred consecutive outpatients with PD were administered an Italian version of the QUIP-RS and a brief neuropsychological assessment to evaluate global cognitive status and scales to measure depression, apathy and impulsive disorders. We evaluated the internal consistency, convergent and divergent validity, and factorial structure of QUIP-RS. We also explored the possible association between QUIP-RS scores and clinical factors and dopaminergic medication.

**Results:**

Subsyndromal ICDs manifestations were observed in 54% of the patients, and one in four (22%) reported two or more ICDs or related behaviors. The QUIP-RS demonstrated good internal consistency (Cronbach’s alpha = 0.806) and construct validity, and its factorial structure reflected different ICDs and ICBs domains. No association emerged between QUIP-RS scores and the clinical aspects of PD and dopaminergic medication.

**Conclusion:**

We provided, for the first time, an Italian translation of the QUIP-RS and demonstrated its feasibility in clinical and research settings. Severity of ICDs was independent of clinical factors and dopaminergic medication, underlining the need to adopt a broader perspective on their etiopathology in PD.

**Supplementary Information:**

The online version contains supplementary material available at 10.1007/s10072-024-07304-2.

## Introduction

Impulse control disorders (ICDs) encompass a class of psychiatric disorders described as a failure to resist an impulse or temptation to perform a behavior harmful to either the self or others [1]. ICDs are characterized by a growing sense of tension or activation before committing the act and a sense of pleasure or “release” during and immediately after the act [2].

ICDs occur frequently in Parkinson’s disease (PD), with prevalence rates widely ranging from 3.5 to 42.8% [3–5], which likely reflect methodological differences in study designs, assessment methods (i.e., informant-based or patient-outcome, use of diagnostic interviews or screening questionnaires), and socio-cultural factors that influence the phenomenology of ICDs worldwide [6]. Different subtypes of ICDs have been reported in PD, including compulsive buying, pathological gambling, binge eating, and hypersexuality, along with a range of impulsive-compulsive behaviors (ICBs) such as punding, walkabout, hobbyism, and compulsive dopaminergic medication overuse, the latter also known as dopamine dysregulation syndrome (DDS) [7]. Although ICDs in PD are often explained as side effects of dopamine replacement therapies, especially dopamine agonists (DA) [8], their occurrence in PD (PD-ICD) has also been reported in the early stages of the disease in drug-naive patients [9]. Altered functioning of the mesocorticolimbic network seems to be responsible for ICDs [10, 11], with increased activity in the ventral striatum and orbitofrontal cortex (OFC) and decreased activity in the anterior cingulate cortex (ACC) strongly related to PD-ICD manifestations [12]. Moreover, ICDs are associated with more severe cognitive manifestations, particularly executive dysfunctions [13, 14], supporting the pivotal role of the prefrontal cortex in behavioral regulation in PD patients.

The clinical relevance and psychosocial consequences associated with ICDs strengthen the need for an efficient early detection of these disorders in PD. To fill this gap, Weintraub and colleagues [7] first developed a global screening instrument titled the Questionnaire for Impulsive-Compulsive Disorders in Parkinson’s Disease (QUIP) [15] for the diagnosis and screening of PD-ICD, and subsequently, the Questionnaire for Impulsive-Compulsive Disorders in Parkinson’s Disease - Rating Scale (QUIP-RS) [16], which allows quantification of the severity and evolution of both ICDs and ICBs.

The QUIP-RS has been tested and validated in numerous languages [17–21]; however, no study has provided and validated an Italian version. To this end, the present study aimed to provide and validate an Italian version of the QUIP-RS, providing its psychometric properties and exploring the possible associations between impulsive-compulsive disorders and clinical variables in an Italian PD population.

## Materials and methods

### Participants

One hundred consecutive PD outpatients were recruited from the Movement Disorders Unit of the IDC-Hermitage Capodimonte in Naples. Patients were included in the study if they met the following inclusion criteria: (i) diagnosis of idiopathic PD based on clinical diagnostic criteria [22], (ii) absence of other neurological and psychiatric conditions, and (iii) absence of cognitive impairment as measured by the Montreal Cognitive Assessment (indicated by a score above 15.5) [23].

Demographic variables such as age, sex, and education (i.e., years of formal education), as well as clinical data such as disease duration, stage of disease, and level of functional disability, assessed using the Hoehn and Yahr staging system (H&Y), and severity of motor symptoms assessed by part III of the Unified Parkinson’s Disease Rating Scale (MDS-UPDRS-III) were registered. As for dopaminergic medication, we calculated the total levodopa equivalent daily dose (LEDD) and the use of levodopa, DA, catechol-O-methyl transferase (COMT), and monoamine oxidase-B (MAO-B) alone.

The present study was approved by the Local Ethics Committee and performed according to the ethical standards of the Declaration of Helsinki and its later amendments. Participants provided informed consent, and the data were treated according to the current regulations.

### Patients and methods

Patients were administered a brief behavioral assessment comprising the QUIP-RS, Beck Depression Inventory-II (BDI-II), Dimensional Apathy Scale (DAS), and a modified version of the Minnesota Impulsive Disorders Interview (MIDI).

#### The Italian version of QUIP-RS

The QUIP-RS is a self-completed or rater-administered rating scale developed to measure the frequency and severity of symptoms of four ICDs (pathological gambling, hypersexuality, compulsive buying, and compulsive eating) and three related disorders (hobbyism, punding, and DDS) over the preceding 4 weeks. For each disorder, the scale uses 4 questions rated on a 5-point Likert scale (from “Never” = 0 to “Very often” = 4) to evaluate commonly reported thoughts, urges and desires, difficulty in controlling behaviors, and deceptive behaviors associated with ICDs (e.g., lying, mounting debts, engaging in illegal acts) [16]. Scores for each ICD and related disorder range from 0 to 16 (higher scores indicate greater severity/frequency of symptoms), with a total QUIP-RS score ranging from 0 to 112.

All items comprising the original version of the QUIP-RS [16] were independently translated into Italian by two researchers (G.M. and C.V.), the different versions were compared, and a third arbitrator (G.S.) solved discrepancies to reach an agreement. The draft of the Italian version of the QUIP-RS was back-translated into English by a native English speaker with expertise in linguistics and psychology to test the linguistic and psychological equivalence of the two versions following the guidelines of Beaton et al. [24]. Subsequently, linguistic comprehensibility and readability were assessed and judged as adequate and equivalent to the original version by a preliminary group of 25 individuals (aged 18–65 years), and the translated version was proofread and approved by the developer of the original questionnaire (D.W.). The The Italian version of the QUIP-RS is presented in Supplementary Material 1.

#### Assessment

Patients were administered a modified version of the MIDI, a 36-item semi-structured clinical interview designed to assess the presence of impulsive disorders [25, 26]. Different screening modules are present for each ICD (compulsive buying, pathological gambling, excessive sexual behavior, compulsive eating, and punding behavior), evaluated using a general screening question and subsequent questions to evaluate the presence of ICD according to the DSM-IV-TR criteria [25].

The BDI-II is a 21-item self-report questionnaire developed to measure depressive symptomatology [27]. Participants were required to select the most appropriate statement to describe their mood during the previous 2 weeks. Statements are organized according to the severity of their content on a 4-point scale from a minimum of 0 points to a maximum of 3, with higher scores indicating greater severity of depressive symptoms.

The DAS is a 24-item scale used to measure the multidimensional nature of apathy [28]. It consists of different subscales to assess executive, emotional, and behavioral initiation apathy, with each item rated on a 4-point Likert scale. The DAS total score ranges from 0 to 72, with higher scores indicating more severe apathy.

All questionnaires and QUIP-RS were rater-administered.

### Statistics

Acceptability of the QUIP-RS was defined as appropriate in the absence of missing values, following previous studies on the standardization of behavioral scales [27, 29–31]. Floor and ceiling effects were also analyzed. Non-parametric statistics were used because of the non-normality of QUIP-RS total score, i.e., skewness and kurtosis values exceeding |2|.

Internal consistency was tested via Cronbach’s α coefficient. Principal component analysis (PCA) with direct OBLIMIN rotation was used to evaluate the factorial structure of the scale. We employed the Mineigen criterion (eigenvalues > 1), together with inspection of the scree plot, to determine the number of factors to be extracted.

Convergent validity was assessed between the QUIP-RS and MIDI total scores, while divergent validity was evaluated by the correlation between the QUIP-RS total score and the BDI-II and DAS total scores. The potential association between demographic (i.e., age and educational level) and clinical factors (i.e., UPDRS-III, disease duration, and medication) and total QUIP-RS score was evaluated using Spearman’s correlation. Sex differences in QUIP-RS scores were assessed using the Mann-Whitney *U* test.

Statistical analyses were performed using IBM SPSS Statistics version 26.

## Results

Our final sample comprised 100 PD patients (62% males), aged 67.45 ± 9.63 years with a mean disease duration of 9.91 ± 4.78 (Table 1).

**Table 1:** Descriptive statistics on demographic, clinical, and neuropsychological variables

	Mean ± SD/ number (frequency)
Age (ys)	67.45 ± 9.63
Education (ys)	11.48 ± 4.39
Gender (*n*)	M = 62 (62%); F = 38 (38%)
Disease duration (ys)	9.91 ± 4.78
UPDRS-III	14.61 ± 8.33
Hoehn and Yahr	2.29 ± 0.53
LEDD (mg)	740.33 ± 382.45
Levodopa alone (mg)	493.37 ± 277.75
DA *n* (%)	66 (66%)
DA alone (mg)	145.32 ± 124.99
COMT inhibitors alone (mg)	26.44 ± 95.67
MAO-B inhibitors alone (mg)	73.08 ± 49.96
MoCA	20.46 ± 4.60
BDI-II	9.20 ± 7.46
DAS	24.74 ± 10.76

*SD* standard deviation, *ys* years, *n* number, *UPDRS* Unified Parkinson’s Disease Rating Scale, *LEDD* Levodopa equivalent daily dose, *mg* milligrams, *DA* dopamine agonists, *COMT* catechol-O-methyl transferase, *MAO-B* monoamine oxidase-B, *MoCA* Montreal Cognitive Assessment, *BDI-II* Beck Depression Inventory-II, *DAS* Dimensional Apathy Scale

### QUIP-RS scores in PD population

We considered the QUIP-RS total score for the investigation of floor and ceiling effects; the floor effect (score 0) was observed in 46% of the sample, but no ceiling effect was observed.

**Fig. 1:**
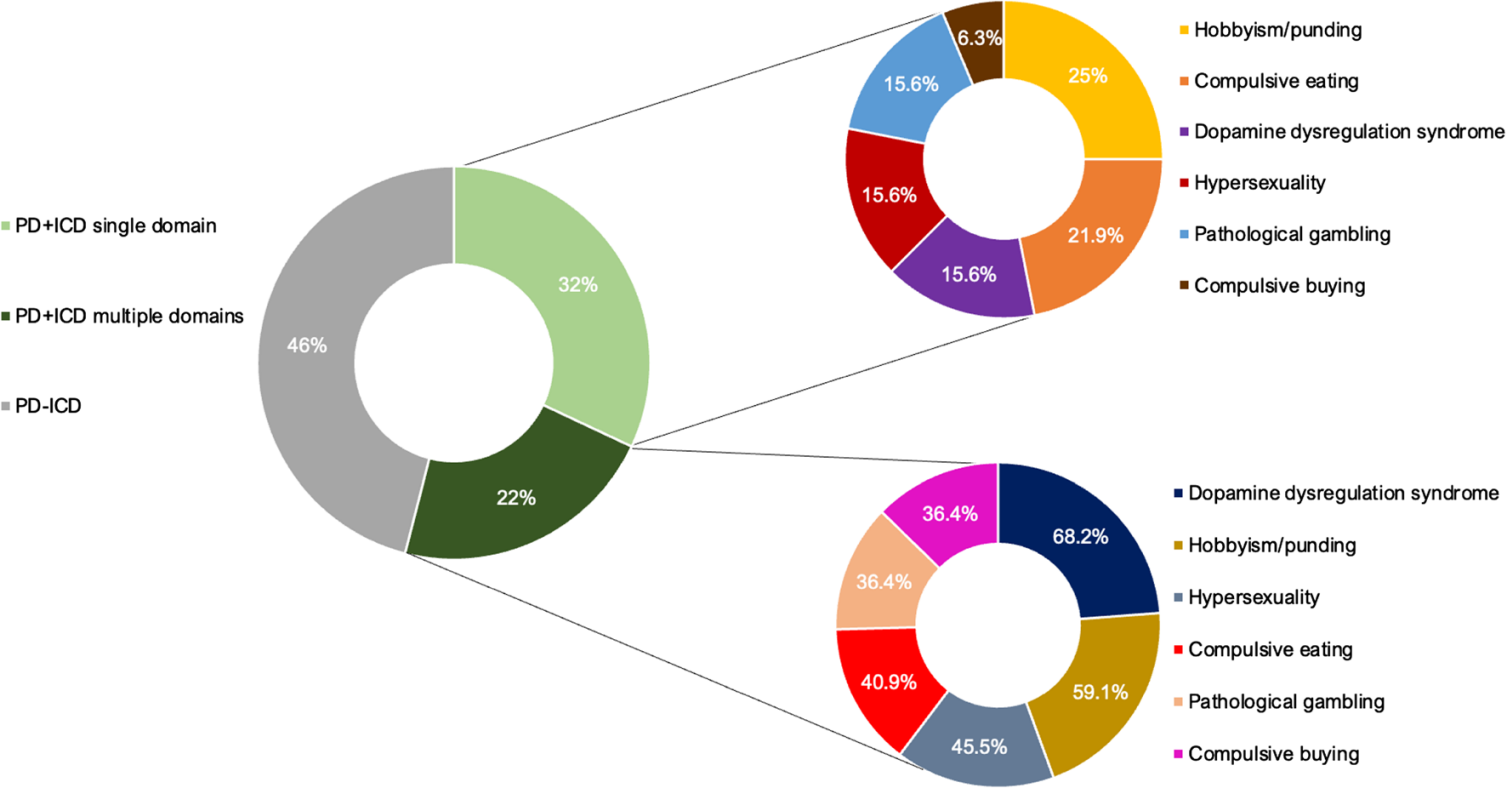
Pie charts displaying percentages of the prevalence of impulse control disorder and related behavior (ICDs) in Parkinson’s disease (PD+ICD) according to the QUIP-RS. In detail, on the right, the frequencies of specific ICDs and related behaviors within PD+ICD single and multiple domains. Note that percentages of specific ICDs and related behaviors within PD+ICD multiple domains chart exceed 100% due to comorbidity

Fifty-four patients were screened as positive (≥ 1, subclinical ICDs and ICD-related disorders) for at least one impulsive-compulsive disorder (54%), and 22% reported two or more ICDs or related behaviors (Fig. 1). Particularly, hobbyism/punding and compulsive eating were the most frequent domains within PD+ICD single domain, whereas DDS was more common within PD+ICD multiple domains (Fig. 1). Considering the whole sample, hobbyism/punding was the most frequently reported symptom (21%), followed by DDS (20%), compulsive eating (16%), hypersexuality (15%), pathological gambling (13%), and compulsive buying (10%). Considering the recommended cut-off scores proposed by Weintraub and colleagues [16], 11 PD patients (11%) were classified as having ICDs, and among these, compulsive eating was observed in 9%, pathological gambling in 6%, hypersexuality in 3%, and compulsive buying in 2%. Regarding ICD-related disorders, 12 patients (12%) reported a score above the cut-off for hobbyism/punding, whereas no cut-off was previously established for the DDS domain.

### Internal consistency and exploratory factor analysis

The QUIP-RS demonstrated good internal consistency (Cronbach’s α = 0.806) (Table 2). A seven-factor solution with an explained variance of 80.55% was generated by PCA with direct OBLIMIN rotation using the Mineigen criterion (eigenvalues > 1; Table 3) and was confirmed by inspection of the scree plot. This structure substantially reflected the items’ classification of different impulsive, compulsive, and related disorders: the first factor loaded under items evaluating hobbyism (explained variance = 18.70%), the second factor included items assessing compulsive eating (explained variance = 15.76%), and the third factor comprised items evaluating compulsive buying (explained variance = 13.43%). The fourth, fifth, sixth, and seventh factors included items assessing pathological gambling (explained variance = 10.96%), hypersexuality (explained variance = 9.07%), use of PD medication (explained variance = 7.57%), and punding (explained variance = 5.06%), respectively. The question of deceptive behaviors associated with punding behavior was initially loaded under the *hobbyism* factor, but it could be placed within the punding factor because of a lower factor based on the suitability of the content.

**Table 2:** Item characteristics of the Italian version of the QUIP-RS

	Mean ± SD	Item-total correlation	Corrected Item-total correlation	Cronbach’s alpha if Item removed
1. How much do you think about the following behaviors (such as having trouble keeping thoughts out of your mind or feeling guilty)?				
Gambling?	0.24 ± 0.73	0.511	0.171	0.807
Sex?	0.28 ± 0.77	0.378	0.316	0.800
Buying?	0.15 ± 0.54	0.484	0.157	0.806
Eating?	0.39 ± 0.99	0.591	0.424	0.795
Performing tasks or hobbies?	0.30 ± 0.82	0.581	0.577	0.787
Repeating simple activities?	0.32 ± 0.95	0.624	0.263	0.804
Taking your PD medications?	0.36 ± 0.85	0.453	0.382	0.797
2. Do you have urges or desires for the following behaviors that you feel are excessive or cause you distress (including becoming restless or irritable when unable to participate in them)?				
Gambling?	0.18 ± 0.72	0.576	0.144	0.808
Sex?	0.21 ± 0.66	0.326	0.325	0.800
Buying?	0.10 ± 0.46	0.593	0.214	0.804
Eating?	0.34 ± 0.93	0.638	0.420	0.795
Performing tasks or hobbies?	0.21 ± 0.74	0.525	0.464	0.794
Repeating simple activities?	0.20 ± 0.72	0.548	0.340	0.799
Taking your PD medications?	0.26 ± 0.75	0.533	0.380	0.797
3. Do you have difficulty controlling the following behaviors (such as increasing them over time, or having trouble cutting down or stopping them)?				
Gambling?	0.12 ± 0.54	0.472	0.204	0.804
Sex?	0.15 ± 0.69	0.581	0.276	0.802
Buying?	0.10 ± 0.46	0.434	0.217	0.804
Eating?	0.30 ± 0.89	0.434	0.340	0.800
Performing tasks or hobbies?	0.23 ± 0.81	0.576	0.503	0.791
Repeating simple activities?	0.24 ± 0.81	0.523	0.386	0.797
Taking your PD medications?	0.15± 0.59	0.583	0.418	0.797
4. Do you engage in activities specifically to continue the following behaviors (such as hiding what you are doing, lying, hoarding things, borrowing from others, accumulating debt, stealing, or being involved in illegal acts)?				
Gambling?	0.20 ± 0.83	0.472	0.139	0.809
Sex?	0.11 ± 0.53	0.472	0.386	0.798
Buying?	0.03 ± 0.22	0.581	0.094	0.806
Eating?	0.13 ± 0.58	0.434	0.410	0.797
Performing tasks or hobbies?	0.09 ± 0.49	0.434	0.421	0.798
Repeating simple activities?	0.05 ± 0.41	0.576	0.388	0.800
Taking your PD medications?	0.15 ± 0.63	0.523	0.290	0.801

*QUIP-RS *Questionnaire for Impulsive-Compulsive Disorders in Parkinson's Disease- Rating Scale, *SD *Standard deviation

**Table 3:** Principal component analysis

	Factor 1	Factor 2	Factor 3	Factor 4	Factor 5	Factor 6	Factor 7
1. How much do you think about the following behaviors (such as having trouble keeping thoughts out of your mind or feeling guilty)?							
Gambling?	−0.092	−0.021	−0.023	**0.834**	0.010	−0.028	0.138
Sex?	−0.026	−0.023	−0.067	0.074	**0.920**	0.062	0.037
Buying?	−0.110	0.093	**−0.896**	0.014	−0.019	0.016	0.109
Eating?	0.021	**0.936**	−0.114	−0.008	0.002	0.009	−0.014
Performing tasks or hobbies?	**0.827**	0.243	0.023	0.018	−0.050	−0.096	0.027
Repeating simple activities?	−0.035	−0.034	0.008	−0.019	−0.031	−0.018	**0.966**
Taking your PD medications?	−0.020	−0.015	−0.015	0.103	0.133	**−0.731**	0.059
2. Do you have urges or desires for the following behaviors that you feel are excessive or cause you distress (including becoming restless or irritable when unable to participate in them)?							
Gambling?	0.069	0.008	0.021	**0.906**	−0.028	0.044	−0.094
Sex?	0.103	−0.042	−0.027	−0.063	**0.963**	0.069	−0.050
Buying?	−0.053	0.133	**−0.947**	0.010	0.020	0.030	0.043
Eating?	0.058	**0.964**	−0.048	−0.018	−0.001	0.017	−0.061
Performing tasks or hobbies?	**0.964**	−0.033	−0.001	−0.014	−0.011	−0.039	−0.036
Repeating simple activities?	0.115	−0.029	−0.006	−0.047	−0.027	−0.003	**0.901**
Taking your PD medications?	0.055	−0.023	−0.032	0.109	0.050	**−0.815**	−0.081
3. Do you have difficulty controlling the following behaviors (such as increasing them over time, or having trouble cutting down or stopping them)?							
Gambling?	0.063	0.004	−0.009	**0.855**	−0.049	−0.052	−0.047
Sex?	−0.018	−0.002	0.055	−0.059	**0.822**	−0.072	−0.036
Buying	0.023	0.051	**−0.964**	0.004	0.041	0.006	−0.004
Eating?	0.010	**0.971**	−0.062	0.020	−0.059	0.163	0.005
Performing tasks or hobbies?	**0.914**	0.033	−0.006	0.001	0.021	−0.057	−0.018
Repeating simple activities?	0.090	−0.008	−0.018	0.037	0.026	0.010	**0.929**
Taking your PD medications?	0.039	0.034	0.008	−0.052	0.028	**−0.903**	−0.021
4. Do you engage in activities specifically to continue the following behaviors (such as hiding what you are doing, lying, hoarding things, borrowing from others, accumulating debt, stealing, or being involved in illegal acts)?							
Gambling?	−0.029	−0.013	0.031	**0.888**	0.028	0.001	−0.020
Sex?	−0.084	0.212	0.062	0.000	**0.425**	−0.368	0.093
Buying?	0.114	−0.163	**−0.739**	−0.044	−0.009	−0.088	−0.117
Eating?	0.000	**0.743**	0.166	−0.047	0.060	−0.244	−0.018
Performing tasks or hobbies?	**0.862**	−0.088	−0.028	−0.009	0.026	0.018	0.057
Repeating simple activities?^§^	**0.686**	−0.031	0.028	0.028	0.066	0.148	**0.261**
Taking your PD medications?	0.015	−0.029	−0.009	−0.070	−0.168	**−0.900**	0.013
Variance explained (%)	18.70	15.76	13.43	10.96	9.07	7.57	5.06
Correlation (*r*) with total score	0.617*	0.527*	0.238	0.524*	0.284	0.463*	0.478*
Cronbach’s α	0.926	0.933	0.905	0.862	0.889	0.897	0.843

Major loadings for each item are displayed in bold

*p < .001

^§^Item placed on a subscale of a lower factor loading based on the better fit with the content

### Validity

Convergent validity was demonstrated by a moderate correlation between the QUIP-RS and MIDI total scores (*r*_s_ = 0.509, *p* < 0.001), whereas divergent validity was demonstrated by the absence of an association between the QUIP-RS total score and the BDI-II (*r*_s_ = 0.129, *p* = 0.240) and DAS total scores (*r*_s_ = −0.084, *p* = 0.445).

### Associations with demographic and clinical factors

The QUIP-RS total score was not associated with age (*r*_s_ = −0.119, *p* = 0.259), educational level (*r*_s_ = −0.046, *p* = 0.669), or sex (*U* = 1143.000, *p* = 0.793). As for clinical variables, the QUIP-RS total score was not related to disease duration (*r*_s_ = −0.071, *p* = 0.537), H&Y stage (*r*_s_ = −0.068, *p* = 0.586), and UPDRS-III (*r*_s_ = 0.070, *p* = 0.590). Considering PD medication, no associations emerged between the QUIP-RS total score and the LEDD total score (*r*_s_ = 0.100, *p* = 0.388), amount of daily levodopa alone (*r*_s_ = 0.229, *p* = 0.102), amount of DA alone (*r*_s_ = 0.059, *p* = 0.606), doses of COMT inhibitors alone (*r*_s_ = 0.126, *p* = 0.373), and doses of MAO inhibitors alone (*r*_s_ = −0.053, *p* = 0.711).

## Discussion

The present study provided the first Italian version of the QUIP-RS and demonstrated its validity and reliability for the assessment of ICDs and related disorders in patients with PD. Fifty-four percent of the patients were screened as positive considering the subsyndromal manifestations, with hobbyism/punding (21%) and DDS (20%) emerging as the most frequent symptoms. Only 11% of the patients were classified as having PD-ICD, according to the recommended cut-off scores [16].

Although the percentages of ICDs such as pathological gambling, compulsive eating, hypersexuality, and compulsive buying partially overlapped with those reported in previous studies [17, 18], we found a higher percentage of patients complaining of ICD-related behaviors such as punding and DDS. The nature of the relationship between hobbyism and punding remains unclear because they may represent a unique neurobiological entity. Patients seem to be aware but unable to reduce the time spent on the execution of repetitive, excessive, and non-goal-directed behaviors and become irritable when distracted from them, possibly leading to isolation from or conflict with other people [32, 33]. Punding has been associated with a pattern of compulsive medication overuse defined by Giovannoni [34] as the inability to stop dopaminergic replacement therapy (DRT) intake to alleviate motor impairment, which instead produces intoxication and severe dyskinesias. Although 20% of patients obtained a score ≥ 1 in the DDS domain of the QUIP, most complained of frequent thoughts (18%) and urges and desires (13%) to take medication rather than a pattern of pathological use and overdosing of DRT (reported by 7% of patients), which is essential for the definition of DDS [34]. Therefore, it is possible to hypothesize that the high percentage of patients with DDS does not reflect an actual high prevalence of this comorbidity that, instead, can be due to anxiety-related mechanisms driven by off-period dysphoria [35] and then triggered by patients’ perception that the “ON” phase is fading away.

Furthermore, the Italian version of the QUIP-RS demonstrated good internal consistency, and a seven-factor structure was revealed by factor analysis reflecting seven ICD domains: hobbyism, compulsive eating, compulsive shopping, DDS, pathological gambling, punding, and hypersexuality. The only exception in the partition of items into factors was represented by the item assessing the engagement in activities for maintaining punding behavior that loaded into the hobbyism factor. However, this result further confirms the hypothesis that hobbyism and punding may be combined to form a single diagnosis [16].

Convergent and divergent validity were demonstrated by our correlational results, revealing a strong positive association of the QUIP-RS with the MIDI and the absence of a relationship with scales assessing depression and apathy. Indeed, the MIDI was originally developed for use in adults and represents a valuable interview tool for ICDs in clinical and research settings [26]; however, it neglects some ICD-related manifestations, such as hobbyism and DDS, whose assessment has been included in the QUIP-RS, designed to cover the whole range of ICDs and related behaviors reported in PD [16].

Although some studies have provided evidence of a link between ICDs and neuropsychiatric disorders set on the opposite extreme of the motivated behavior continuum, such as apathy and depression, these disorders may rarely co-occur [36, 37], that may explain the absence of relationships in our study.

Interestingly, the QUIP-RS score was independent of the PD-related processes and DRT. Although some studies have shown that ICDs are more prevalent in young unmarried male subjects and in patients with a longer disease duration [38] as an adverse effect of DA use in PD [39], recent findings have shown that the mere contribution of DA intake and other clinical factors does not fully explain the pathophysiology and prognosis of ICDs in PD [8, 40, 41]. First, it should be noted that the current clinical approach in planning DRT posology promotes the use of extended-release formulations that allow the maintenance of more stable drug concentrations and lower DA dosages to minimize the risk of ICD development and subsequent medical-legal issues [8, 40, 42]. In addition, personality profiles characterized by impulsiveness and novelty seeking, or a family history of substance use disorders, represent risk factors for the development of ICDs [43]. At the same time, biological sex may also influence the development of specific ICDs with male patients being more prone to hypersexuality and eating disorders, whereas compulsive shopping is more common among female patients [38]. Finally, it should be considered that some types of ICDs are also affected by cultural, religious, and socioeconomic factors (e.g., pathological gambling is likely to be less prevalent or underreported in India and China, as public gambling or casino visiting is not a socially accepted habit in these countries) [38]. Taken together, these findings suggest adopting a broader perspective on the etiopathology of ICDs, whose occurrence seems to result from the interaction between personality traits, sex, cultural factors, neuropathological processes, and DRT impact [8, 40, 41].

However, the present study presents some limitations. First, the QUIP-RS was administered to patients, which can limit our results because patients may be unaware of the clinical significance of their symptoms or may hide or not report undesirable behaviors due to embarrassment or sociocultural factors leading to underdiagnosis and undertreatment [44, 45]. Future studies should also ascertain the presence of ICDs using an informant version of the QUIP-RS to avoid delays in diagnosis and therapeutic interventions [45]. Moreover, further studies are needed to determine the inter-rater and test-retest reliability of the Italian version of the QUIP-RS and to investigate its sensitivity to symptom changes after therapeutic interventions.

In conclusion, this study demonstrated that the Italian version of the QUIP-RS represents the most appropriate alternative for the evaluation of ICDs in parkinsonian syndromes compared to other behavioral assessments previously available within the Italian scenario [46]. Indeed, it has proven to be a comprehensive, reliable, and valid screening instrument for ICDs and related behaviors, and its adoption is recommended in PD population for both research and clinical purposes.

### Supplementary information

ESM 1 (DOCX 22 kb)

## Data Availability

Datasets associated with the present study are available upon reasonable request of interested researchers.
