# Supplementary material for: Validation of the Italian version of the Questionnaire for Impulsive-Compulsive Disorders in Parkinson’s Disease-Rating Scale (QUIP-RS) in an Italian Parkinson’s disease cohort
Source: Neurol Sci. 2024 Jan 17;45(7):3153–61. doi: 10.1007/s10072-024-07304-2 (PMC11176207; doi:10.1007/s10072-024-07304-2)
Supplement: Supplementary file 1 — (DOCX 22 kb) [file 10072_2024_7304_MOESM1_ESM.docx]

**Questionnaire for Impulsive-Compulsive Disorders in Parkinson’s Disease – Rating Scale**

**(QUIP-RS)**

Traduzione Italiana a cura di Maggi et al.

**Somministrato a: ______Paziente _______Caregiver _______Paziente e Caregiver**

**Paziente / Soggetto: _____________________________________**

**Data di somministrazione: _______________________________**

1. Quanto spesso ha pensieri relativi ai seguenti comportamenti (quanto, ad esempio, ha difficoltà a mantenere lontani dalla sua mente questi pensieri o quanto, ad esempio, si sente in colpa)?

| Gioco d’azzardo? | __Mai(0) | __Raramente(1) | __Qualche volta(2) | __Spesso (3) | __Molto spesso(4) |
| --- | --- | --- | --- | --- | --- |
| Sesso? | __Mai(0) | __Raramente(1) | __Qualche volta(2) | __Spesso (3) | __Molto spesso(4) |
| Fare acquisti? | __Mai(0) | __Raramente(1) | __Qualche volta(2) | __Spesso (3) | __Molto spesso(4) |
| Alimentazione? | __Mai(0) | __Raramente(1) | __Qualche volta(2) | __Spesso (3) | __Molto spesso(4) |
| Svolgimento di attività o hobby? | __Mai(0) | __Raramente(1) | __Qualche volta(2) | __Spesso (3) | __Molto spesso(4) |
| Ripetizione di attività semplici? | __Mai(0) | __Raramente(1) | __Qualche volta(2) | __Spesso (3) | __Molto spesso(4) |
| Assunzione di farmaci specifici per la Malattia di Parkinson? | __Mai(0) | __Raramente(1) | __Qualche volta(2) | __Spesso (3) | __Molto spesso(4) |

1. Sente la necessità o prova il desiderio di mettere in atto i seguenti comportamenti che lei percepisce come eccessivi o fonte di stress (incluso diventare irrequieto o irritabile quando non ha la possibilità di realizzarli)?

| Gioco d’azzardo? | __Mai(0) | __Raramente(1) | __Qualche volta(2) | __Spesso (3) | __Molto spesso(4) |
| --- | --- | --- | --- | --- | --- |
| Sesso? | __Mai(0) | __Raramente(1) | __Qualche volta(2) | __Spesso (3) | __Molto spesso(4) |
| Fare acquisti? | __Mai(0) | __Raramente(1) | __Qualche volta(2) | __Spesso (3) | __Molto spesso(4) |
| Alimentazione? | __Mai(0) | __Raramente(1) | __Qualche volta(2) | __Spesso (3) | __Molto spesso(4) |
| Svolgimento di attività o hobby? | __Mai(0) | __Raramente(1) | __Qualche volta(2) | __Spesso (3) | __Molto spesso(4) |
| Ripetizione di attività semplici? | __Mai(0) | __Raramente(1) | __Qualche volta(2) | __Spesso (3) | __Molto spesso(4) |
| Assunzione di farmaci specifici per la Malattia di Parkinson? | __Mai(0) | __Raramente(1) | __Qualche volta(2) | __Spesso (3) | __Molto spesso(4) |

1. Ha difficoltà a controllare i seguenti comportamenti (come ad esempio la tendenza ad aumentarne la frequenza nel tempo oppure la difficoltà a ridurre o interrompere uno dei seguenti comportamenti)?

| Gioco d’azzardo? | __Mai(0) | __Raramente(1) | __Qualche volta(2) | __Spesso (3) | __Molto spesso(4) |
| --- | --- | --- | --- | --- | --- |
| Sesso? | __Mai(0) | __Raramente(1) | __Qualche volta(2) | __Spesso (3) | __Molto spesso(4) |
| Fare acquisti? | __Mai(0) | __Raramente(1) | __Qualche volta(2) | __Spesso (3) | __Molto spesso(4) |
| Alimentazione? | __Mai(0) | __Raramente(1) | __Qualche volta(2) | __Spesso (3) | __Molto spesso(4) |
| Svolgimento di attività o hobby? | __Mai(0) | __Raramente(1) | __Qualche volta(2) | __Spesso (3) | __Molto spesso(4) |
| Ripetizione di attività semplici? | __Mai(0) | __Raramente(1) | __Qualche volta(2) | __Spesso (3) | __Molto spesso(4) |
| Assunzione di farmaci specifici per la Malattia di Parkinson? | __Mai(0) | __Raramente(1) | __Qualche volta(2) | __Spesso (3) | __Molto spesso(4) |

1. Si impegna in attività con lo scopo di mantenere in atto i seguenti comportamenti (come ad esempio nascondere quello che sta facendo, mentire, accumulare cose, chiedere prestiti, accumulare debiti, rubare, o essere coinvolti in azioni illegali)?

| Gioco d’azzardo? | __Mai(0) | __Raramente(1) | __Qualche volta(2) | __Spesso (3) | __Molto spesso(4) |
| --- | --- | --- | --- | --- | --- |
| Sesso? | __Mai(0) | __Raramente(1) | __Qualche volta(2) | __Spesso (3) | __Molto spesso(4) |
| Fare acquisti? | __Mai(0) | __Raramente(1) | __Qualche volta(2) | __Spesso (3) | __Molto spesso(4) |
| Alimentazione? | __Mai(0) | __Raramente(1) | __Qualche volta(2) | __Spesso (3) | __Molto spesso(4) |
| Svolgimento di attività o hobby? | __Mai(0) | __Raramente(1) | __Qualche volta(2) | __Spesso (3) | __Molto spesso(4) |
| Ripetizione di attività semplici? | __Mai(0) | __Raramente(1) | __Qualche volta(2) | __Spesso (3) | __Molto spesso(4) |
| Assunzione di farmaci specifici per la Malattia di Parkinson? | __Mai(0) | __Raramente(1) | __Qualche volta(2) | __Spesso (3) | __Molto spesso(4) |

Traduzione Italiana della QUIP-RS a cura di Maggi et al.

English original Version 1.0 (7/01/09) Copyright © University of Pennsylvania 2009: University of Pennsylvania holds the copyright to the QUIP-RS and QUIP, so a licensing agreement with Penn is required for its use in any language, which is without cost for academic use.

**Questionnaire for Impulsive-Compulsive Disorders in Parkinson’s Disease – Rating Scale**

**(QUIP-RS)**

Traduzione Italiana a cura di Maggi et al.

**Soggetto: _________________________________________________**

**Data di valutazione: _________________________________________________**

**SCORING**

| **A.** | **Gioco d’azzardo patologico** | **_________** | **(0-16)** |
| --- | --- | --- | --- |
| **B.** | **Ipersessualità** | **_________** | **(0-16)** |
| **C.** | **Shopping compulsivo** | **_________** | **(0-16)** |
| **D.** | **Alimentazione incontrollata** | **_________** | **(0-16)** |
| **E.** | **Hobby-Punding** | **_________** | **(0-32)** |
| **F.** | **Sovradosaggio di farmaci dopaminergici** | **_________** | **(0-16)** |

**Totale Punteggio ICD (A-D) _________ (0-64)**

**Totale Punteggio QUIP-RS (A-F) _________ (0-112)**

Traduzione Italiana della QUIP-RS a cura di Maggi et al.

English original Version 1.0 (7/01/09) Copyright © University of Pennsylvania 2009: University of Pennsylvania holds the copyright to the QUIP-RS and QUIP, so a licensing agreement with Penn is required for its use in any language, which is without cost for academic use.
